# Genome-wide gene-based analysis suggests an association between Neuroligin 1 (*NLGN1*) and post-traumatic stress disorder

**DOI:** 10.1038/tp.2016.69

**Published:** 2016-05-24

**Authors:** V Kilaru, S V Iyer, L M Almli, J S Stevens, A Lori, T Jovanovic, T D Ely, B Bradley, E B Binder, N Koen, D J Stein, K N Conneely, A P Wingo, A K Smith, K J Ressler

**Affiliations:** 1 Department of Psychiatry and Behavioral Sciences, Emory University School of Medicine, Atlanta, GA, USA; 2 School of Life Sciences, Manipal University, Manipal, India; 3 Department of Human Genetics, Emory University, Atlanta, GA, USA; 4 Mental Health Service Line, Department of Veterans Affairs Medical, Atlanta, GA, USA; 5 Department of Translational Research in Psychiatry, Max Planck Institute of Psychiatry, Munich, Germany; 6 Department of Psychiatry, University of Cape Town, Cape Town, South Africa; 7 Department of Psychiatry and MRC Unit on Anxiety and Stress Disorders, University of Cape Town, Cape Town, South Africa; 8 McLean Hospital, Harvard Medical School, Belmont, MA, USA

## Abstract

Post-traumatic stress disorder (PTSD) develops in only some people following trauma exposure, but the mechanisms differentially explaining risk versus resilience remain largely unknown. PTSD is heritable but candidate gene studies and genome-wide association studies (GWAS) have identified only a modest number of genes that reliably contribute to PTSD. New gene-based methods may help identify additional genes that increase risk for PTSD development or severity. We applied gene-based testing to GWAS data from the Grady Trauma Project (GTP), a primarily African American cohort, and identified two genes (*NLGN1* and *ZNRD1-AS1*) that associate with PTSD after multiple test correction. Although the top SNP from *NLGN1* did not replicate, we observed gene-based replication of *NLGN1* with PTSD in the Drakenstein Child Health Study (DCHS) cohort from Cape Town. *NLGN1* has previously been associated with autism, and it encodes neuroligin 1, a protein involved in synaptogenesis, learning, and memory. Within the GTP dataset, a single nucleotide polymorphism (SNP), rs6779753, underlying the gene-based association, associated with the intermediate phenotypes of higher startle response and greater functional magnetic resonance imaging activation of the amygdala, orbitofrontal cortex, right thalamus and right fusiform gyrus in response to fearful faces. These findings support a contribution of the *NLGN1* gene pathway to the neurobiological underpinnings of PTSD.

## Introduction

Post-traumatic stress disorder (PTSD) is a complex psychiatric disorder that affects 7–8% of the US population.^1, 2^ While exposure to a traumatic event is necessary for the development of PTSD, only a fraction of trauma-exposed individuals develop this condition,^2^ suggesting that some people may be more susceptible than others. Studies estimate that 30–70% of the variation in PTSD risk can be attributed to genetic factors,^3, 4^ and several studies have identified individual variants associated with PTSD.^5^ Identification of genetic variants that increase risk for PTSD may facilitate the development of diagnostic, as well as therapeutic strategies.^6^

Genome-wide association studies (GWAS) have successfully led to the identification of common single nucleotide polymorphisms (SNPs) that increase the risk of PTSD development in an individual.^7^ The first GWAS for PTSD^8^ reported an association between a SNP in retinoid-related orphan receptor alpha (*RORA*) and PTSD. A larger GWAS of PTSD did not replicate the association with *RORA* but did identify an association with a SNP in a long intergenic non-coding RNA.^9^ Similarly, three other GWAS of PTSD^10, 11, 12^ identified associations with SNPs in *PRTFDC1* and intergenic regions. However, replication of GWAS associations across PTSD studies remains a limiting factor.

GWAS results are often difficult to replicate in individual cohorts, potentially because of differences in power, linkage disequilibrium (LD) structure, genotyping coverage or clinical characteristics of a cohort. As an alternative, gene-based association tests are well-suited to identify genes that may increase susceptibility to complex diseases like PTSD. They complement traditional GWAS and have helped identify additional genes associated with complex traits in previous studies.^13, 14, 15^ Gene-based associations are more likely to replicate across populations, in part because they are able to aggregate signals across variants and have a lower threshold for multiple test correction.^16, 17^ Different methods for gene-based association testing have been proposed, many of which are optimally powered to detect associations under a variety of conditions.^18^ Several commonly used methods have recently been implemented in FAST,^19^ a software program that allows for concurrent gene-based testing of a dataset. The goal of this current study was to use gene-based association testing to identify and characterize genes that associate with PTSD in two independent cohorts. In addition, we examined the PTSD intermediate phenotypes of neural response to fearful faces^20, 21^ and startle reflex^22, 23^ in the identified genes.

## Materials and methods

Note that the different cohorts, sizes, genotyping platforms and additional materials about the cohorts are provided in Supplementary Table 2.

### Grady Trauma Project

The subjects in this cohort were part of a larger investigation of genetic and environmental factors that predict the response to stressful life events in a predominantly African American, urban population of low socioeconomic status.^24^ Research participants were approached in the waiting rooms of primary care clinics of a large, public hospital. After the subjects provided written informed consent, they participated in a verbal interview and gave a saliva and/or blood sample for genetic analyses.

Lifetime traumatic life events were assessed via a semi-structured interview using the Traumatic Events Inventory (TEI), a scale developed by the GTP researchers during their prior work in the Grady hospital primary care population. The TEI considers experiencing, witnessing or being confronted with traumatic events such as natural disaster, serious accident or injury, sudden life-threatening illness, being in war zone, physical assault, sexual assault, witnessing violence between parents or caregivers as a child, and childhood abuse. The higher the TEI score, the more traumatic life events one encountered. The TEI has been used in several of our published work.^25, 26, 27^

Current PTSD was assessed using the modified PTSD Symptom Scale (PSS), an 18-item self-report scale with excellent internal consistency, high test-retest reliability and concurrent validity to diagnose PTSD consistent with DSM-IV criteria.^28, 29^ As such, a subject was considered to have current PTSD if he/she had at least one symptom in the re-experiencing cluster, three symptoms in the avoidance and numbing cluster, and two symptoms in the hyperarousal cluster, and the duration of these symptoms were at least 1 month following a traumatic experience.^30^ The PTSD cases in our analysis had current PTSD symptoms while the controls did not. Both the cases and controls had been exposed to at least one traumatic life experience. Since the PSS assesses current PTSD symptoms, we cannot rule out that some of the controls might have had PTSD in the past. All procedures were approved by the Institutional Review Board of Emory University School of Medicine and the Grady Health Systems Research Oversight Committee.

DNA was extracted from saliva in Oragene collection vials (DNA Genotek, Ottawa, ON, Canada) using the DNAdvance kit (Beckman Coulter Genomics, Danvers, MA, USA), while DNA from blood was extracted using either the E.Z.N.A. Mag-Bind Blood DNA Kit (Omega Bio-Tek, Norcross, GA, USA) or ArchivePure DNA Blood Kit (5 Prime, Gaithersburg, MD, USA). Genotyping was performed using the Omni-Quad 1M or the Omni Express BeadChip (Illumina, San Diego, CA, USA), and genotypes were called in Illumina’s GenomeStudio (Illumina). PLINK^31^ was used to perform quality control measures. Initial quality control involved removing samples with very low call rates (that is, poor quality samples with high amounts of missing data) and outside acceptable levels of heterozygosity (−0.25<Fhet>0.25), and the remaining samples were re-called in GenomeStudio.^32^ Further quality control involved excluding SNPs that had a call rate <98%, a minor allele frequency (MAF) <0.01 or significant deviation from Hardy–Weinberg proportions (*P<*1 × 10^−6^ in controls and *P<*1 × 10^−10^ in PTSD cases), and excluding samples with >2% missing data. We further identified and removed related individuals by using PLINK to estimate the proportion of identity by descent (IBD) for each pair of individuals. Among pairs of individuals with an IBD proportion>0.12 (indicating cousins or a closer relation), we removed the individual in each pair with the higher rate of missing genotype data. We used PLINK to prune the autosomal data in windows of 50 bp, removing 1 SNP from each pair of SNPs with *r*^2^>0.05 to obtain a set of roughly independent markers (~50 000 SNPs). We note that the pruned dataset was only used to calculate the principal components but not for gene-based association testing. Principal-component analysis (PCA) at autosomal SNPs not in LD was then performed to infer axes of ancestry and remove outlier subjects. Based on PCA, we retained those African American subjects who fell within 3 s.d. of the medians of the first and second PCs in our sample. Approximately 4000 subjects were considered for this study, of which detailed phenotype information was available for 3678 subjects.

PLINK was used to regress the PTSD status on allele count assuming an additive model (0, 1 or 2 copies of risk allele), including sex, chip type (Omni-Quad 1M or the Omni Express BeadChip) and the top 10 PCs calculated from GWAS data as covariates.^33, 34^ Gene-based association tests were performed using the FAST software using the summary data derived from the SNP-based association tests described above.^19^ minSNP calculates a gene-based *P*-value either directly from a parametric distribution or by using the permuted *P*-value of the best individual SNP association within the given gene. minSNP computes single-SNP F-statistics for each SNP within a gene and uses the best F-statistic within that gene as its test statistic, which is then converted to a *P*-value with gene-based permutations to correct for gene size.^18^ BIMBAM is a Bayesian association method that calculates and averages the Bayes factors for all the SNP models within a given gene to compute the test statistic.^35^ BIMBAM results are similar to those obtained from the minSNP method as the sum of the Bayes factor is dominated by the single best SNP. VEGAS uses the sum of *X*^2^ for an individual SNP to generate a test statistic suggested for the gene. The *P*-value of the gene is then computed after accounting for LD and the number of SNPs in each gene.^17^ GWiS identifies a subset of SNPs that maximizes the model probability. The model is essentially a subset of SNPs within a gene with *p* total SNPs that are permitted to have non-zero regression coefficients, and the test statistic is calculated as the posterior model probability approximation.^18^ Gates is a Simes’ test ^36^ modification that computes the *P*-value based on the formula:


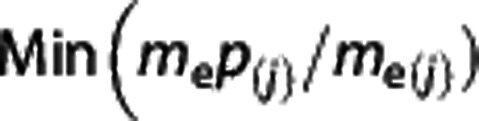


where, *m*_e(*j*)_ is the effective number of independent *P*-values among the top *j* SNPs and *m*_e_ is the effective number of independent *P*-values among the *m* SNPs. This method estimates association while accounting for the effective number of independent *P*-values among the top SNPs.^16^

Each gene-based test is likely to perform better based on different genetic architectures (multiple independent signals versus single signal in a gene, size of gene, patterns of LD in the gene and so on). For example, GWiS is better powered when genes house multiple independent causal variants than if each gene has a single causal variant. On the other hand, minSNP and BIMBAM perform better when fewer SNPs underlie a specific trait. VEGAS can be biased towards identification of causal variants in LD blocks represented by many SNPs, as opposed to blocks with fewer SNPs, and performs best in the absence of LD. VEGAS also loses power if the MAF is low, whereas GWiS, minSNP and BIMBAM remain unaffected.^18^ Since each method is substantially different from the other, FAST provides a platform to run several gene-based tests concurrently, thereby enabling the identification of significant associations using the best performing test for a given genomic region.

The pre-computed haplotype and index files for the ASW population were downloaded from the FAST wiki page and used as a reference. The number of tests performed for each method was calculated based on the number of genes analyzed (*N*=32 194), and a Bonferroni correction was applied to account for multiple testing. All *P*-values reported are two-sided.

### Drakenstein Child Health Study

The DCHS cohort consisted of pregnant women with a high degree of trauma exposure. These pregnant women were recruited from primary health care clinics in the Drakenstein sub-district of the Cape Winelands, Western Cape, South Africa, between March 2012 and June 2013.^37^ Participants were enrolled from the Mbekweni and TC Newman clinics in Drakenstein. While Mbekweni served a primarily black African population, the TC Newman clinic served a multiethnic population. These at-risk women were assessed longitudinally for trauma-related psychiatric disorders.^38^ Assessment of lifetime traumatic events was carried out in a semi-structured interview, in which participants were asked if they had experienced any of the following: fire/explosion, car/train crash, other unintentional injury, interpersonal violence, weapon threat, rape, attempted rape, other sexual assault, illness/injury, witnessed unintentional injury, witnessed violence, birth trauma/pregnancy trauma, death of a loved one or other traumatic experiences in their life time. All participants gave informed consent prior to participating in the study, which was approved by the Institutional Review Board of the University of Cape Town, South Africa.

PTSD was assessed with the modified PSS^29^ in the prenatal period and with the Mini International Neuropsychiatric Interview (MINI)^39^ at 6 to 18 months postpartum. While the PSS assesses current PTSD, the MINI, which is a structured clinician-administered diagnostic interview for DSM-IV psychiatric disorders, assesses both current and lifetime PTSD. Hence, both cases and controls of lifetime PTSD had been exposed to at least one traumatic life event in their life, and while the cases manifested PTSD symptoms, the controls did not. In DCHS, 380 subjects had available genotypes and clinical information. We did not include participants without lifetime traumatic experiences in our analysis.

DNA was extracted from whole blood using the QIAsymphony DSP DNA Midi kit and protocol (Qiagen, Hilden, Germany). Genome-wide SNP genotyping was conducted using the Illumina Infinium PsychArray Beadchip (Illumina). Standard quality control of the genome-wide data was performed using PLINK^31^ removing individuals with >5% missing data and removing one in each pair of related individuals with an IBD proportion >0.12 (indicating cousins or a closer relation). We removed SNPs with call rates <95%, MAF <0.05 and deviation from Hardy–Weinberg proportions (*P<*1 × 10^−6^ in controls and *P<*1 × 10^−10^ in PTSD). To evaluate population stratification, PC eigenvectors of the genetic relationship matrix were calculated by using about 50 000 independent SNPs using the same method as the GTP sample with the following exceptions: SNPs associated with psychiatric disease (that is, the custom content on the PsychArray, 50 000 SNPs), and SNPs in LD (*r*^2^=0.075) were excluded to calculate PCs. Dimensional plots of the PCs were also used to remove outliers. The Tracy–Widom Statistic was used to evaluate the number of PCs used in the analyses, and the first PC was found to be sufficient to account for population structure. Logistic regression was applied to evaluate the relationship between lifetime PTSD diagnosis and each SNP, adjusting for PCs as covariates.

### Replication of PTSD-associated genes

Any genes surviving multiple test correction in GTP were tested in DCHS. We note that the replication phase consisted of focused testing of PTSD-associated genes with only the gene-based method associated in the discovery phase: *NLGN1* with minSNP and *ZNRD1-AS1* with Vegas.

### eQTL identification

The genotype and expression data for four brain tissues (cerebellum, frontal cortex, temporal cortex and pons) were downloaded from GEO (GSE15745) and dbGAP (phs000249.v1.p1). Genotypes within 50 kb of *NLGN1* were imputed with MACH, using the European Phase 1 1000 Genomes data as a reference. Imputed SNPs with an estimated *r*^2^<0.3 between imputed and true genotypes and those with posterior probabilities <0.9 for the most likely genotype were excluded from subsequent analysis. For each brain region, *NLGN1* genotypes were tested for association with *NLGN1* expression using linear regression models.

### Brain imaging acquisition and analysis

To determine the effects of *NLGN1* on brain function, we used rs6779753 (the most associated SNP) to represent the gene-based association signal for GTP. The effect of *NLGN1* rs6779753 genotype on brain responses to fearful faces was measured in 53 trauma-exposed female participants drawn from the larger GTP cohort, using functional magnetic resonance imaging (fMRI). These 53 participants comprised of 20 PTSD cases (8 GG, 11 TG and 1 TT) and 33 controls (12 GG, 17 TG, and 4 TT). Participants viewed blocks of either fearful or neutral face stimuli, with eight face trials per block. Trials were composed of a 500-ms fixation cross, followed by a 500-ms face stimulus presentation. This task has been described in detail previously^20, 40, 41, 42^ and is available in the Supplementary Methods

For each participant, hemodynamic responses to blocks of fearful and neutral stimuli were modeled with a boxcar function representing the onset and 8000-ms duration of the block, convolved with a canonical hemodynamic response function, as implemented in SPM8 (Wellcome Trust Centre for Neuroimaging, University College of London, London, UK). Participant-specific motion parameters were included as covariates. Contrast images representing the linear comparison of beta values for the fearful versus neutral conditions were constructed for each participant, and were entered into group-level random effects analysis to identify clusters of significant activation. Group-level models also included the first two ancestry PCs from genome-wide data. No other demographic variables were included as covariates, as there were no significant differences between the genotype groups.

The effects of genotype on brain activation were assessed in a hypothesis-independent manner, voxel-wise across the whole brain. Task-based functional connectivity analyses were conducted using the CONN toolbox (http://web.mit.edu/swg/software.htm). Seed regions were defined anatomically using the mean time course across voxels within right and left amygdala regions of interest, defined anatomically using the SPM Anatomy Toolbox.^43^ For each voxel within the whole-brain mask, covariance with amygdala activation during responses to fearful face stimuli was contrasted with covariance with amygdala activation during responses to neutral faces. Motion parameters and main effects of task condition were modeled as subject level covariates, and the first two ancestry PCs from genome-wide data were included as group-level covariates. For both regional activation and functional connectivity analyses, an additive model (zero, one or two copies of the T allele) was used to examine genotype associations.

Analyses of regional activation and functional connectivity were examined using a combined height-extent threshold to correct for multiple comparisons. Monte Carlo simulation was implemented using AlphaSim within the REST toolbox for SPM8,^44^ for voxels within a gray-matter mask based on the ICBM152 template.

The neuroimaging analyses included a careful correction for multiple comparisons. Analyses of both activation and connectivity were conducted voxel by voxel across the whole brain, rather than targeting *a priori* regions of interest. Multiple testing correction was implemented using the Alphasim method, estimating a combined height-extent threshold for significant clusters. This represents a ‘principled’ approach to controlling the family-wise error rate in neuroimaging studies that allows for similar control of type 1 error across studies (Bennett *et al.*, 2009, SCAN),^45^ and is currently a very widely used method for statistical correction of multiple comparisons. All findings reached a corrected threshold of *P<*0.05.

### Startle response measurements

The psychophysiological data were acquired using Biopac MP150 for Windows (Biopac Systems, Aero Camino, CA, USA) on a subset of the GTP cohort. This GTP subset was comprised of 352 individuals, which included 126 PTSD cases (59 GG, 60 GT and 7 TT) and 208 controls (85 GG, 90 GT and 33 TT). Phenotype information on PTSD diagnosis was unavailable for 18 (10 GG, 7 GT and 1 TT) of the 352 individuals. All data were sampled at 1000 Hz and amplified with a gain of 5000 using the electromyography (EMG) module of the Biopac system. The acquired data were filtered, rectified and smoothed using MindWare software (MindWare Technologies, Gahanna, OH, USA) and exported for statistical analyses. The EMG signal was filtered with low- and high-frequency cutoffs at 28 Hz and 500 Hz, respectively. The eyeblink component of the acoustic startle response was measured by EMG recordings of the right orbicularis oculi muscle with two 5-mm Ag/AgCl electrodes filled with electrolyte gel. One electrode was positioned 1cm below the pupil of the right eye and the other was 1cm below the lateral canthus. Impedance levels were <6 kΩ for each participant. The startle probe was a 108-dB (A) SPL, 40-ms burst of broadband noise with 0 rise time, delivered through headphones. The maximum amplitude of the eyeblink muscle contraction 20–200 ms after presentation of the startle probe was used as a measure of startle magnitude. The startle data were collected as part of a fear conditioning experiment which consisted of a habituation phase followed by three blocks of conditioning that occurred without any breaks.

The habituation phase contained startle probes and shapes that were later paired with the unconditioned stimulus (US) during the conditioning phase. The US was a 250-ms airblast with an intensity of 140 ψ directed to the larynx. All reinforced conditioned stimuli (CS+) trials were paired with the US, while the non-reinforced CS trials were never paired with the US. Both CSs were 6 s in duration. In all phases of the experiment, inter-trial intervals ranged from 9 to 22 s. The association between startle magnitude and rs6779753 genotypes (GG, GT, TT) was examined using an analysis of variance in which age, sex, race, trauma history and PTSD symptom severity were used as covariates.

## Results

The GTP and DCHS cohorts had comparable rates of PTSD (31.5% and 35.2%, respectively) with 1158 cases and 2520 controls in GTP, and 134 cases and 246 controls in DCHS. The GTP analysis was restricted to African American ancestry, as indicated by the GWAS, while the DCHS cohort was more diverse with both black African and mixed populations. Another key difference between the two cohorts is that the DCHS cohort is exclusively female, while GTP includes ~30% male participants. While the mean age was ~40±13 years in GTP, DCHS subjects were younger (26.4±5.6 years). There were no significant differences in age or trauma exposure between PTSD cases and controls in each cohort.

### Gene-based association

**Figure 1:**
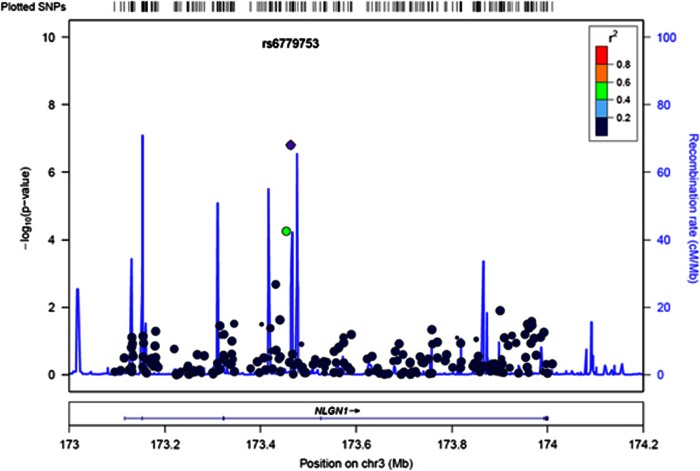
Regional association plot for all *NLGN1* SNPs in GTP. The plot was created using GWAS association data that served as the input for FAST. The *x*-axis represents the distribution of SNPs across the gene while the *y*-axis represents the –log_10_ of the *P*-value of each SNP in the gene. The colors indicate the *r*^2^ between the SNP with the lowest *P*-value and all the other SNPs. GTP, Grady Trauma Project; GWAS, genome-wide association studies; SNP, single nucleotide polymorphism.

Gene-based association tests were run for the GTP cohort using FAST, and the results from each of the five methods are summarized in Table 1 and Supplementary Figure 1. The minSNP method identified an association between *NLGN1* (*P=*1.00 × 10^−6^) and PTSD that remained after Bonferroni correction for multiple testing. This approach takes the lowest *P*-value of any SNP in *NLGN1,* and calculates a parametric *P*-value for the gene based on 1 000 000 permutations. For the GTP cohort, the SNP with the lowest *P*-value in *NLGN1* was rs6779753 (Figure 1). The VEGAS method identified an association between *ZNRD1-AS1* (*P=*1.00 × 10^−6^) and PTSD, which remained after Bonferroni correction. This method calculates the sum of individual SNP associates to generate a test statistic for the gene that accounts for LD and the number of SNPs with a given gene.^17^ No other method identified associations between any genes and PTSD after multiple test correction, but it is interesting to note that four of the five gene-based approaches used, including GWiS (*P=*1.47 × 10^−5^), BIMBAM (*P=*2.04 × 10^−5^) and Gates (*P=*2.16 × 10^−5^), identified *NLGN1* as the gene most associated with PTSD (Table 1).

To replicate these findings, *NLGN1* and *ZNRD1-AS1* were evaluated in the DCHS cohort, using the minSNP and VEGAS methods, respectively. *NLGN1* associated with PTSD in DCHS (*P*=0.0015; Supplementary Figure 2). However, for DCHS, the SNP most associated with PTSD was rs4894661, and rs6779753 did not associate (*t*=0.28, *P*=0.78). On calculating LD (Supplementary Figure 3), we found that the most associated SNPs for GTP (rs6779753) and DCHS (rs4894661) were not in LD and thus gave independent signals in each cohort. Neither imputation of additional SNPs in *NLGN1* and *ZNRD1-AS1* in DCHS nor limiting the replication analysis to the subset of black South Africans (*N*=205) resulted in association of rs6779753. However, *NLGN1* was significantly associated with PTSD in both the imputed and race-specific analyses (*P<*0.05). *ZNRD1-AS1,* on the other hand, did not replicate its association with PTSD in DCHS (*P*=0.267).

### Regulation of *NLGN1* in the brain

**Figure 2:**
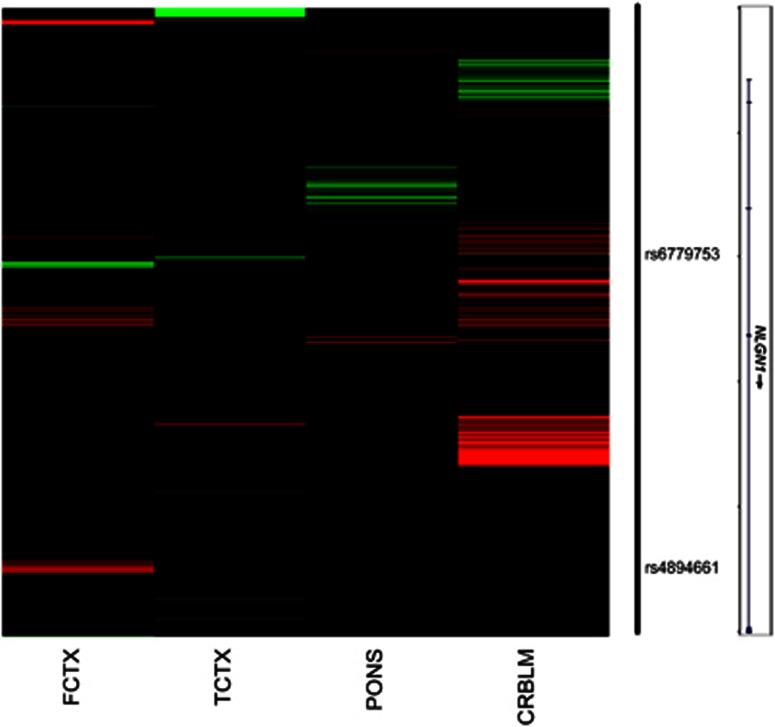
Association of *NLGN1* SNPs with expression in the brain. Heat map of the *t*-statistic for all the SNPs that associate with *NLGN1* expression in the frontal cortex (FCTX), temporal cortex (TCTX), pons (PONS) and cerebellum (CRBLM). SNPs are oriented by position. A *t*-statistic greater than 1.98 is colored in red, indicating positive association of the major allele with expression levels, while a *t*-statistic lesser than −1.98 is colored in green. SNP, single nucleotide polymorphism.

Since association between *NLGN1* and PTSD was replicated in an independent cohort at the gene-based level, subsequent analyses focused on possible influences of *NLGN1* genotype on brain pathways implicated in PTSD. We first evaluated the association between genetic variation in *NLGN1* and gene expression in postmortem samples from all four brain regions available in a Caucasian cohort from publically available GEO and dbGAP databases. Overall, *NLGN1* SNPs associated with gene expression in each of the brain regions (Figure 2), but the direction of association varied by region such that a genotype may associate with lower expression in the frontal cortex and higher expression in the temporal cortex. Examination of rs6779753, the SNP that most associated with PTSD in GTP, showed that the TT genotype associated with lower *NLGN1* expression in the cerebellum (*P*=0.044) but not in the other tissues evaluated. Interestingly, examination of the *NLGN1* SNP with the strongest association in DCHS, rs4894661, indicated that the risk genotype (CC) associates with lower expression in the frontal cortex (*P*=0.010) but not in the other tissues.

### Effects of *NLGN1* genotype on regional activation and functional connectivity

**Figure 3:**
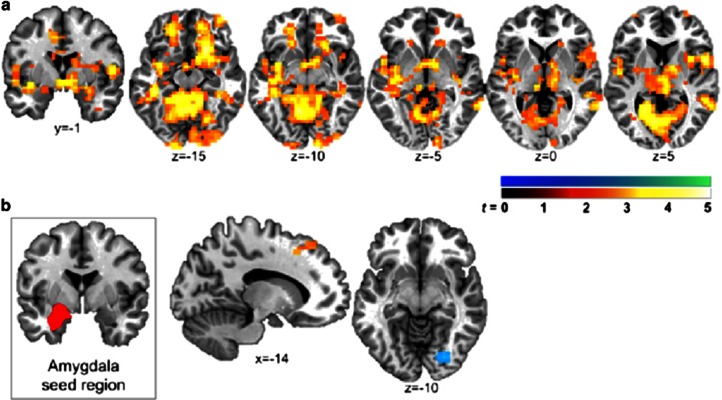
Effect of *NLGN1* genotype on BOLD response to threat stimuli (fearful>neutral faces). This analysis was conducted on a subset of 53 GTP participants (20 PTSD cases and 33 controls. (**a**) Regions that showed a significant linear association with the number of *NLGN1* risk alleles, *P<*0.05, corrected. No region showed a decrease in activation associated with the number of risk alleles. (**b**) Effect of *NLGN1* polymorphism on functional connectivity with the amygdala, *P<*0.05, corrected. Results are shown for the left amygdala seed region; *NGLN1* polymorphism did not influence right amygdala connectivity. Images are shown in neurological orientation, overlaid on a single-subject template in Montreal Neurological Institute (MNI) space. PTSD, post-traumatic stress disorder.

We next considered the potential effects of *NLGN1* and brain function in probable PTSD patients. In the GTP cohort, 53 trauma-exposed participants had both genetic data and fMRI data. Using rs6779753 to represent gene-based association signal for GTP, we examined the association between genotype and brain responses to threat (fearful versus neutral face stimuli), as an intermediate phenotype underlying PTSD. Whole-brain analysis showed that the number of T alleles associated with greater activation in limbic and prefrontal regions including bilateral amygdala, bilateral orbitofrontal cortex and the right thalamus, as well as regions associated with face processing such as the right fusiform gyrus (Figure 3a, Supplementary Table 1).

We then tested for regions in which amygdala connectivity associated with threat (fearful>neutral) correlated with the number of T alleles. As shown in Figure 3b, the number of T alleles was associated with increased connectivity between the left amygdala seed and left dorsolateral prefrontal cortex (dlPFC; in the superior frontal gyrus, *x*,*y*,*z*=−14, 16, 46, *Z*=2.82, *k*=19), and decreased connectivity between left amygdala and right fusiform gyrus (*x*,*y*,*z*= 26, −80, −10, *Z*=3.35, *k*=21). *NLGN1* genotype did not associate with right amygdala connectivity.

### Effect of *NLGN1* genotype on startle response

**Figure 4:**
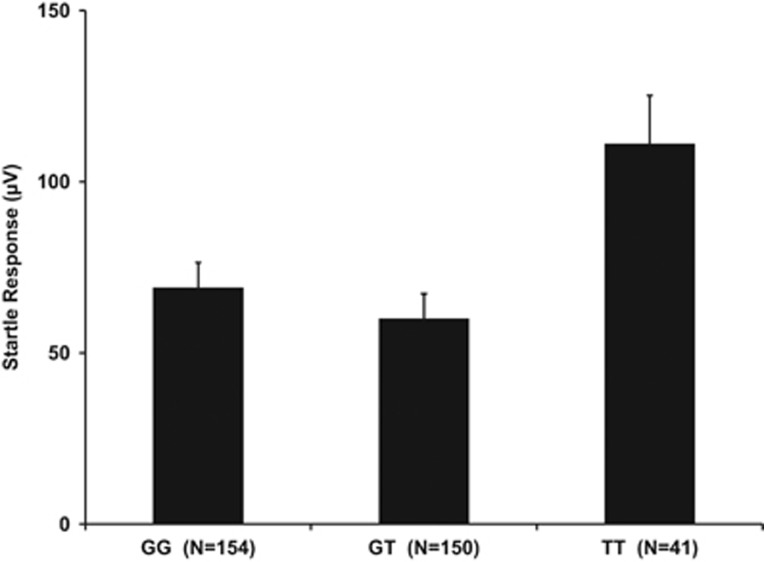
Association of *NLGN1* genotype and startle response. This analysis was conducted on a subset of 352 GTP participants (126 PTSD cases and 208 controls). The *y-*axis represents the mean+s.e. of the magnitude of the startle response (in μV) during the habituation phase across the three rs6779753 genotypes (*x*-axis = GG, GT, TT). GTP, Grady Trauma Project; PTSD, post-traumatic stress disorder.

The auditory fear-potentiated startle reflex also serves as a well-replicated intermediate phenotype for PTSD.^46^ Thus, we examined startle reactivity in 352 individuals from GTP using EMG of the eyeblink muscle prior to and during fear conditioning. The startle analysis included 352 individuals, of which 126 were PTSD cases (59 GG, 60 GT and 7 TT) and 208 were controls (85 GG, 90 GT and 33 TT). Phenotype information on PTSD diagnosis was unavailable for 18 of the 352 individuals. We found a significant main effect of genotype on magnitude of the startle response to the 108 dB white noise probe prior to conditioning (F_(2,349)_=5.23; *P*=0.006). *Post hoc* tests showed that the startle response was greater in the TT genotype compared with both GT (*P*=0.001) and GG (*P*=0.008) individuals (Figure 4). The differences remained significant after controlling for age, sex, race, degree of child and adult trauma, and PSS score (F_(2,320)_=6.03; *P*=0.003).

To address whether the above finding may be confounded by case/control status, we further examined startle reactivity in only the 208 individuals who had experienced trauma but did not meet criteria for PTSD diagnosis, using EMG prior to and during fear conditioning. In this smaller group of traumatized controls, we also found a significant main effect of genotype on magnitude of the startle response to the 108 dB white noise probe prior to conditioning (F_(2,207)_=5.09; *P*=0.007, Supplementary Figure 4). *Post hoc* tests showed that the startle response was greater in the TT genotype compared with both GT (*P*=0.002) and GG (*P*=0.05) individuals. These data suggest that those with the TT genotype showed higher reactivity and higher anticipatory anxiety even before the conditioning experiment and independent of PTSD case status.

## Discussion

Although GWAS has identified genetic variants associated with PTSD,^7, 47^ the results are often difficult to replicate in independent cohorts. New gene-based methods may help identify additional genes that increase risk for PTSD development or severity. This study applied a gene-based approach to identify an association between *NLGN1* and PTSD that remained significant after a stringent multiple test correction and replicated in an independent cohort. The SNP that most strongly associated with PTSD in the GTP cohort (rs6779753) was not the SNP most strongly associated in the DCHS cohort (rs4894661). Moreover, the two SNPs were not in LD (Supplementary Figure 2), suggesting each SNP was responsible for an independent signal. This may be due to differences in power or ancestry between the cohorts. It may also be attributable to differences in gender make-up and in methodology for determining PTSD diagnoses across the cohorts. Nonetheless, finding a gene-based association with *NLGN1* across two distinct PTSD cohorts led us to examine possible associations between these genetic variants and underlying intermediate phenotypes.

To further characterize these *NLGN1* variants, we wished to determine if they may be associated with differential gene regulation. *NLGN1* plays an important role in mediating the formation and maturation of synapses in the mammalian brain.^48, 49, 50^ We evaluated the association between genetic variation in *NLGN1* and its expression in postmortem samples from four brain tissues (cerebellum, frontal cortex, temporal cortex and pons) in a Caucasian cohort. Numerous *NLGN1* SNPs associated with its expression in one or more of the four brain regions. For example, the SNP with the strongest association in GTP (rs6779753) and DCHS (rs4894661) associated with *NLGN1* expression only in the cerebellum or frontal cortex, respectively. However, in each case, the PTSD-associated genotype correlated with lower *NLGN1* expression in its respective brain region. Interestingly, *NLGN1* knockout mice exhibit behavioral changes linked to cerebellar and orbitofrontal cortex abnormalities.^51, 52^ Together these data suggest that variants within a gene associated with learning and memory function across cerebellar and cortical areas in mice are also associated with decreased expression and putatively decreased synaptic function, in similar areas in subjects with PTSD.

We next considered the effects of *NLGN1* on brain function related to PTSD in participants who had experienced trauma drawn from the GTP sample. The number of rs6779753 T alleles associated with increased response to fearful stimuli across many brain regions involved in emotional responses and fear. SNP-associated regional activation included the amygdala, orbitofrontal cortex and right thalamus. In addition, the number of T alleles was associated with increased connectivity between the left amygdala and left dlPFC. In tasks probing emotional responses, dlPFC activation has been associated with cognitive regulation of emotional responses, in part via modulation of amygdala activity.^53, 54^ In addition, the dlPFC has been implicated in anticipation or attention to emotional judgment,^55, 56^ suggesting that the connectivity between the amygdala and dlPFC may be important for emotional response and regulation. Our group has previously found increased dlPFC activation to threatening stimuli during an attention bias task in individuals with PTSD relative to traumatized controls,^57^ suggesting that dorsolateral prefrontal function during threat processing may contribute to PTSD symptoms. The current findings point to a possible genetic contributor to changes in amygdala–prefrontal connectivity.

The number of rs6779753 T alleles also associated with decreased connectivity between the left amygdala and right fusiform gyrus. The fusiform gyrus has been implicated in identifying faces and perceiving facial expressions,^58^ and its activation has a modulatory effect on amygdala responses.^59^ Since the amygdala is involved in the emotional aspects of face processing and is activated by perception of fearful expressions in faces,^60^ a decrease in connectivity with the fusiform gyrus may reflect altered perceptual processing of the face stimuli in the fMRI task. Taken together, the functional connectivity findings suggest that the rs6779753 polymorphism influences both regulatory ‘top-down’ and perceptual ‘bottom-up’ circuits regulating emotional reactivity.

Startle reactivity was also evaluated in the GTP cohort, and the TT genotype of rs6779753 associated with higher reactivity and higher anticipatory anxiety, independent of trauma exposure or PTSD symptoms. These results suggest that variation in *NLGN1* may predispose individuals to higher levels anxiety and fear, potentially increasing their risk to develop PTSD following a traumatic event. The startle reflex is generated by the pons,^61^ and the high expression of *NLGN1* in this brain region may underlie the gene-related exacerbation of the startle response.

NLGN1 is localized in excitatory synapses and plays an important role in learning and memory. Long-term potentiation and long term depression in the amygdala associate with the formation and extinction of fear memory, respectively.^62, 63, 64, 65, 66^ In the amygdala, *NLGN1* is essential for the storage of associative fear memory in fear conditioning tasks, and the depletion of *NLGN1* can result in a deficit in fearful memory storage.^67, 68^ NMDA functioning is known to be important for normal emotional and cognitive functioning, and NMDAR levels depend on *NLGN1.* In fact, systemic administration or intra-amygdala infusion of d-Cycloserine (DCS), a partial NMDA agonist, has been found to facilitate the extinction of conditioned fear,^69, 70, 71, 72, 73^ making it a potential treatment option for individuals suffering from the symptoms of PTSD. Placebo-controlled trials have shown that DCS is effective in the treatment of PTSD symptoms^74, 75, 76, 77^ and in reducing startle response in PTSD patients.^78^ DCS is a partial agonist of NMDAR, whose levels depend on *NLGN1*, and DCS not only modulates NMDAR activation,^79^ but also facilitates long-term potentiation.^80, 81^ Thus, these findings suggest that the association of *NLGN1* with PTSD could be related to dysregulation of emotional learning processes, which may underlie trauma associations and failure to recover following trauma exposure.

Studies have also implicated the association of the *NLGN1* gene with other psychiatric disorders like autism spectrum disorders and schizophrenia.^82, 83^ Reduced excitatory synaptic transmission could be a promising mechanism and treatment target for behavioral abnormalities.^51^ Approaches to enhancing NMDA function may be particularly effective in those with low NLGN1 expression in the brain.

This study had some limitations. One was that the DCHS replication cohort was smaller than GTP and differed from it in terms of ancestry, gender composition and clinical assessment of PTSD. It was also run on a different genotyping platform. In GTP, current PTSD was assessed using the PSS scale, therefore unlike in DCHS, we cannot rule out the possibility that some controls had prior PTSD. However, despite the heterogeneity in terms of clinical and demographic characteristics of these cohorts, the results independently support the association of *NLGN1* with PTSD. This variation also suggests that these findings may be generalizable and that genetic variation in *NLGN1* may contribute to PTSD in other cohorts. Also, the analysis of intermediate phenotypes in GTP was conducted in a partially overlapping group of subjects as the discovery cohort. Another limitation is the lack of standardized methods for multiple test correction across the different gene-based methods. However, the results of the different gene-based methods were reasonably correlated, each supporting association of *NLGN1* with PTSD using different approaches. Finally, amygdala tissue would have been most informative for identifying associations between *NLGN1* genotype and expression levels relevant to PTSD. Though data from the amygdala was not available, expression results in other regions of the brain support the functional implications of genetic variation in *NLGN1*.

In conclusion, using a genome-wide, unbiased gene-based approach, we have identified the *NLGN1* gene to be associated with PTSD in two different civilian traumatized cohorts. *NLGN1* has been associated with a variety of psychiatric disorders and may be directly related to synaptic function and synaptic plasticity. Understanding molecular correlates of stress- and fear-related disorders offers hope for future novel, targeted approaches to treating and possibly preventing the sequelae that follow trauma exposure.

## Figures and Tables

**Table 1:** Gene-based association results in the discovery sample (GTP).

*Method*	*# Nominally significant genes (*P*<0.05)*	*# Bonferroni corrected significant genes (*P*<1.55 × 10*^−*06*^)	*Top gene*
BIMBAM	1263	0	*NLGN1*
VEGAS	1145	1	*ZNRD1-AS1*
Gates	1573	0	*NLGN1*
GWiS	194	0	*NLGN1*
minSNP	11 787	1	*NLGN1*

Abbreviation: GTP, Grady Trauma Project; PTSD, post-traumatic stress disorder.

The table lists the number of genes associated with PTSD at the nominal *P*-value <0.05 for each method, as well as the number of genes that remain associated with PTSD following a Bonferroni correction for multiple testing.
