# Supplementary material for: Genome-wide gene-based analysis suggests an association between Neuroligin 1 (NLGN1) and post-traumatic stress disorder
Source: Transl Psychiatry. 2016 May 24;6(5):e820–. doi: 10.1038/tp.2016.69 (PMC5070067; doi:10.1038/tp.2016.69)
Supplement: Supplementary Information [file tp201669x1.doc]

**Supplemental Methods**

*Brain imaging acquisition and analysis*

Brain imaging data were acquired on a Siemens 3.0-Tesla Magnetom Trio TIM MRI scanner (Siemens, Malvern, PA) using a 12-channel head coil. Functional images were acquired using the Z-SAGA pulse sequence ^5^ to minimize signal loss due to susceptibility artifacts. Each scan volume contained 30 axially acquired 4mm thick images with an in-plane resolution of 3.44 x 3.44 mm^2^ utilizing the parameters: pulse repetition time 3000 ms, echo time 1= 30 ms, echo time 2 = 67 ms, at a flip angle of 90 deg. Structural images were acquired using a gradient-echo, T1-weighted pulse sequence (TR=2600ms, TE=3.02ms; 1mm×1mm×1mm voxel size).

Preprocessing and statistical analysis was conducted in SPM8 (Wellcome Department of Cognitive Neurology). ArtRepair software ^6^ was used to correct signal spike and motion artifacts. Slices containing spike artifacts were identified and replaced using linear interpolation, with no more than 5% of slices repaired per participant. Volumes affected by motion artifact were repaired using linear interpolation, with no more than 5% of volumes repaired per participant. Slice timing correction and spatial realignment were applied to the functional images. A 128Hz high-pass filter was used to remove low-frequency noise ^7^. The anatomical image was then co-registered to the mean functional image and normalized to the International Consortium for Brain Mapping (ICBM) 152-subject template. The resulting normalization parameters were applied to the functional images. The functional images were then smoothed with an 8mm Gaussian kernel.

**Supplemental Table 1. Regions in which threat response (Fearful > Neutral) was associated with *NLGN1*** genotype

| **MNI coordinates** | | | | | | |
| --- | --- | --- | --- | --- | --- | --- |
| **Region** | **HEM** | **x** | **y** | **z** | **Z** | **k** |
| *Positive correlation with number of T-alleles* | | | | | | |
| Mid. Cingulate G. | L | -10 | -28 | 26 | 5.09 | 4439 |
| Amygdala | R | 22 | 8 | -26 | 4.51 |  |
| Thalamus | R | 2 | -24 | 14 | 4.45 |  |
| Inf. Temporal G. | R | 62 | -32 | -30 | 4.47 | 82 |
| Inf. Temporal G. | R | 50 | -28 | -30 | 3.31 |  |
| Fusiform G. | R | 42 | -24 | -22 | 3.23 |  |
| Sup. Frontal G. | R | 10 | 60 | 18 | 3.71 | 283 |
| Mid. Frontal G. | L | -38 | 52 | 18 | 3.56 |  |
| Sup. Frontal G. | R | 22 | 60 | 22 | 3.52 |  |
| Orbitofrontal Cortex | L | -22 | 44 | -10 | 3.5 | 105 |
| Orbitofrontal Cortex | L | -18 | 40 | -18 | 3.3 |  |
| Orbitofrontal Cortex | L | -22 | 28 | -10 | 3.14 |  |
| Orbitofrontal Cortex | R | 42 | 56 | -14 | 3.17 | 29 |
| Orbitofrontal Cortex | R | 42 | 44 | -18 | 2.81 |  |
| Temporal Pole | L | -34 | 12 | -34 | 3.01 | 21 |
| Temporal Pole | L | -26 | 8 | -38 | 2.38 |  |
| *Negative correlation with number of T-alleles* | | | | | | |
| * No significant clusters | | | | | | |

Note. All clusters exceeded a corrected threshold of *p*<.05. “HEM” = hemisphere.

**Supplementary Table 2.**  **This Table summarizes the populations, # of cases and controls, genotyping chips, number of variants, and whether imputation was performed.**

| **Analysis** | **Dataset** | **Race** | **#Cases/#Controls** | **# of Variants** | Imputation (Yes/No) |
| --- | --- | --- | --- | --- | --- |
| GWAS/Gene-Based Association | Grady Trauma Project (GTP) | African American | 1158/2520 | 634854^a^ | No |
| Replication - GWAS/Gene-Based Association | Drakenstein Child Health Study (DCHS) | Black African and Multiethnic | 134/246 | 273846^b^ | No^d^ |
| Startle Analysis | Grady Trauma Project (GTP) | African American | 126/210 | 1^a^ | No |
| Brain Imaging Analysis | Grady Trauma Project (GTP) | African American | 20/33 | 1^a^ | No |
| eQTL Analysis | Gibbs et al. (GSE15745) | Caucasian | NA | 2684^c^ | Yes^e^ |

^a^ Illumina OmniQuad 1M and OmniExpress BeadChip

^b^ Illumina Infinium PsychArray Beadchip

^c^ Illumina Infinium HumanHap550 beadchip

^d^ No, but an additional analysis was carried out after the imputation of additional SNPs within NLGN1 and ZNRD1-AS1

^e^ Yes, using the European Phase 1 1000 Genomes dataset as reference

**Supplementary Figure 1. Concordance among results for different gene-based association methods.** Rank orders of the genes for each of the 5 gene-based methods implemented in FAST are depicted in the heatmap. Gates, Vegas and BIMBAM all resulted in similar rank orders, and minSNP had reasonable overlap with those 3 methods, particularly among the genes with the lowest ranks (most significant). GWiS varied the most when compared to the other methods, but still had similarity among the lowest ranking genes, including the top-ranked gene, *NLGN1*.

**Supplementary Figure 2. Regional association plot for *NLGN1* SNPs common between GTP and DCHS.** The x-axis represents the distribution of SNPs across the gene while the y-axis represents the –log_10_ of the p-value of each SNP in the gene. The colors indicate the r^2^ between the SNP with the lowest p value and all the other SNPs. The plots were created using GWAS association data that served as the input for FAST. Though rs9842389 (p=0.014) appears to be the most significant SNP, rs4894661 (p=0.021) was identified as the minSNP after several permutations.


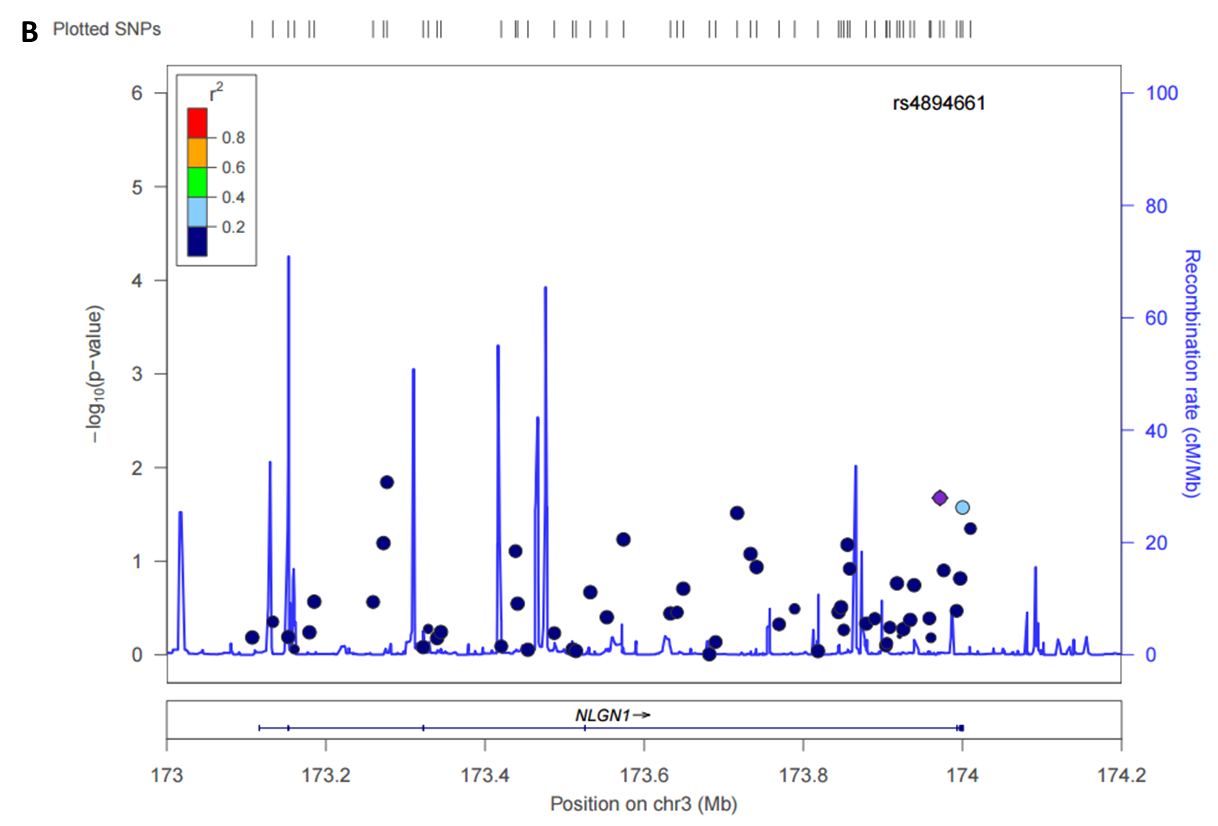


**Supplementary Figure 3. LD patterns (r^2^) among A) all the *NLGN1* SNPs in GTP and B) the *NLGN1* SNPs common between GTP and DCHS.** LD between SNPs is measured as r^2^ with the values indicated within the diamonds. r^2^=0 is shown as white, 0 < r^2^ <1 is shown in gray and r^2^ = 1 is shown in black.

**Supplementary Figure 4. Association of *NLGN1* genotype and startle response in Traumatized Controls Only.**  To control for whether the effect shown in Figure 4 of *NGLN1* genotype on startle reactivity, we also examined the genotype effect only in the traumatized control cohort without PTSD, (control), compared to those with PTSD, demonstrating a similar significant effect as with the combined population.

**
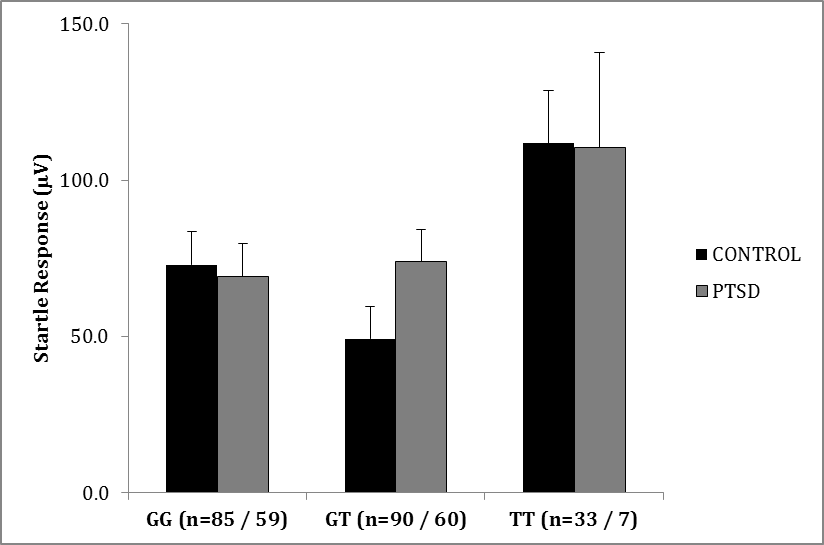
**
